# Hip Joint Osteochondroma: Systematic Review of the Literature and Report of Three Further Cases

**DOI:** 10.1155/2014/180254

**Published:** 2014-05-20

**Authors:** Asim M. Makhdom, Fan Jiang, Reggie C. Hamdy, Thierry E. Benaroch, Martin Lavigne, Neil Saran

**Affiliations:** ^1^Division of Orthopaedic Surgery, Shriners Hospital for Children, Montreal Children Hospital, McGill University, 1529 Cedar Avenue, Montreal, QC, Canada H3G 1A6; ^2^Department of Orthopaedic Surgery, King Abdulaziz University, Jeddah 21589, Saudi Arabia; ^3^Division of Orthopaedic Surgery, Maisonneuve-Rosemont Hospital, University of Montreal, 5415 Assomption Boulevard, Montreal, QC, Canada H1T 2M4

## Abstract

The aim of this study is to systematically review the literature with regards to surgical treatment of patients with hip joint osteochondromas, and to report our surgical management of three paediatric patients who had femoral neck or acetabular osteochondromas in association with acetabular dysplasia. We performed a systematic review using PubMed and Embase databases for all studies that reported surgical treatments for patients with peritrochanteric or acetabular osteochondroma with or without acetabular dysplasia. We also retrospectively reviewed three patients who were diagnosed with a hip osteochondroma in association with actetabular dysplasia. These patients were known to have hereditary multiple exostoses (HME). The systematic review revealed 21 studies that met our inclusion criteria. All studies were case reports and retrospective in nature and failed to conclude a uniform treatment plan. The three reported cases illustrate successful excision of hip osteochondromas and treatment of acetabular dysplasia. Early excision of hip osteochondromas might prevent acetabular dysplasia in HME patients. Routine radiographic pelvic survey at the time of diagnosis of HME is recommended for early detection of hip osteochondromas and acetabular dysplasia in these children.

## 1. Introduction

Osteochondromas are benign osteocartilaginous primary tumours of long bones typically found in the forearm, knees, or ankles [1]. They commonly involve the metaphysis and can cause significant deformities, restriction of range of motion (ROM), persistent pain, and growth disturbance [1–3]. They may occur as a solitary lesion or as multiple lesions in the context of hereditary multiple exostoses (HME), an autosomal dominant disorder with an approximate prevalence of 1 in 50,000 in the general population [4, 5]. Several studies in the literature have reported the occurrences of these lesions in the hip and acetabulum [6–25]. Acetabular dysplasia and coxa valga occur in approximately 25% of HME patients [5]. It has been suggested that acetabular dysplasia and femoral neck osteochondromas may independently or synergistically contribute to the increased risk of lateral subluxation of the hip [26]. Typically, surgical intervention is considered when these features are present. However, the surgical management for such lesions remains challenging for orthopaedic surgeons as they are not commonly encountered in clinical practice. The primary aim of this study is to systematically review the literature with regards to the surgical treatment of patients with hip osteochondromas. The secondary aim is to present our surgical management for three paediatric patients who had hip subluxation secondary to femoral neck/acetabular osteochondromas in association with acetabular dysplasia.

## 2. Materials and Methods

The systematic review was performed using PubMed and Embase databases. Our search terms included “hip osteochondroma,” “proximal femoral osteochondroma,” “femoral neck osteochondroma,” and “acetabular osteochondroma” as well as “acetabular dysplasia” in combination with the previously mentioned terms and “osteochondroma acetabular dysplasia.” The inclusion criteria were all articles that included patients with proximal femur/acetabular osteochondroma with or without acetabular dysplasia and who underwent surgical excision. Exclusion criteria included the following: (1) inadequate description of surgical treatments, (2) articles published in abstract form only, and (3) nonrelevance to the subject of interest. Our goal was to explore the surgical treatments as well as the reported complications in the literature.

In the authors' center, between the year of 2000 and 2011, three patients were diagnosed with hip osteochondromas in association with acetabular dysplasia. After approval from our Institutional Review Board, these three cases were retrospectively reviewed. There were two females and one male and all were known to have HME. The left hip was affected in all patients. Clinical data and basic demographics are summarized in Table 1.

## 3. Results

### 3.1. Literature Review

Our initial search revealed 163 articles found in the PubMed and Embase databases. After removing 20 duplicated articles, 143 articles were reviewed and retained for analysis. Of these, 122 articles were excluded leaving 21 articles meeting the eligibility criteria for our study. All of the articles were case reports and were retrospective in nature (Tables 2–4).

### 3.2. Case Presentation

**Case 1**

**Figure 1:**
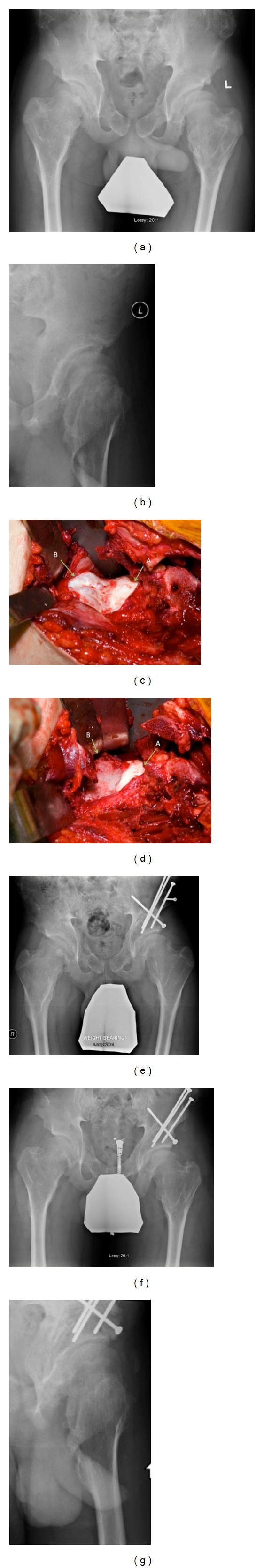
(a) Preoperative anteroposterior pelvic radiograph. (b) Preoperative false profile view showing poor anterior femoral head coverage. (c) Intraoperative images showing the location of the femoral head (arrow A) and the femoral neck osteochondroma (arrow B). (d) Postpartial excision of osteochondroma (arrow B) and the location of the femoral head (arrow A). (e) Anteroposterior pelvic radiograph at 1-year follow-up. (f) Anteroposterior pelvic radiograph at 18 months of follow-up. (g) False profile pelvic radiograph at 18 months of follow-up showing improved anterior coverage.

This fifteen-year-old male known for HME presented with left hip pain with prolonged walking and sporting activities. He underwent multiple previous surgeries (left tibia, right distal femur, and upper extremities) for excision of osteochrondromas. On examination, a Trendelenburg gait was noted. The ROM was restricted in terms of hip abduction and flexion. Pelvic radiographs showed left hip dysplasia (center edge angle (CEA) of 18 degrees) and left femur neck osteochondroma causing left hip subluxation (Figures 1(a) and 1(b)). The surgical procedure was planned aiming to prevent further hip subluxation, relieve his symptoms, and to reduce the risk of osteoarthritis of the left hip in the future. The treating surgeon (N.S) has performed a left hip Bernese periacetabular osteotomy and femoral neck osteoplasty with partial excision of the osteochondroma through a modified Smith-Peterson approach (Figures 1(c) and 1(d)). The patient was kept nonweight bearing on the left lower extremity for 6 weeks and ROM exercises were initiated. At one-year follow-up, the ROM improved significantly and the patient reported no pain. Nevertheless, he had discomfort around the surgical site secondary to a prominent left iliac screw (Figure 1(e)). Therefore, this screw was removed in the operating room. At eighteen months of follow-up, the patient had a normal gait and no associated pain. He has returned to all sports including recreational soccer. The radiographs show good femoral head coverage (CEA = 33 degrees) (Figures 1(f) and 1(g)).

**Case 2**

**Figure 2:**
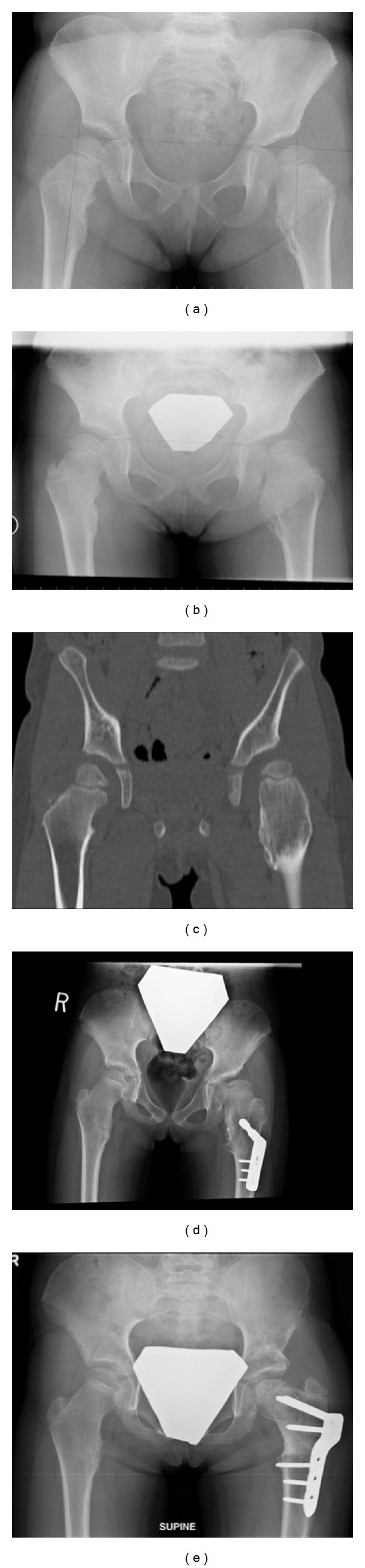
(a) Anteroposterior pelvic radiograph at initial presentation. (b) Anteroposterior pelvic radiograph and (c) Computed tomography of the pelvis at one-year follow-up. (d) Anteroposterior pelvic radiograph 4 years after left femoral varus derotational osteotomy (VDRO) with partial excision of the osteochondroma. (e) Anteroposterior pelvic radiograph ten months after performing the second VDRO, modified Dega osteotomy, and extensive excision of femoral neck osteochondroma. Partial left femoral head necrosis is also noted.

This four-year-old female was referred to the authors' center for a recent diagnosis of HME. On her first visit, she had no complaints and her examination was unremarkable apart from palpable osteochondromas in the upper extremities and distal femora. Radiographs revealed a left femoral neck osteochondroma with bilateral coxa valga (left > right), a left dysplastic hip (CEA 7 degrees), and left hip subluxation (Figure 2(a)). At one year of follow-up, progressive left hip subluxation (CEA = 0) was noted (Figures 2(b) and 2(c)). Consequently, a left femoral varus derotational osteotomy with partial excision of the osteochondroma was performed by the treating surgeon (T.B) through the lateral approach. This was followed by application of a paediatric dynamic hip screw (DHS). The patient was able to walk with a normal gait without any associated pain at 1-year follow-up and by the 2nd year she was able to participate in sports. Her flexion and internal rotation improved significantly on subsequent follow-ups. However, external rotation and abduction of the left hip did not improve. Four years postoperatively, she was noted to have a restricted ROM in terms of hip abduction, external rotation, and flexion. Pelvic radiographs showed significant recurrence of the left hip osteochondroma with persistent left acetabular dysplasia and worsening left hip subluxation (Figure 2(d)). At this time she underwent a proximal femoral varus osteotomy and extensive excision of the left femoral neck osteochondroma through the lateral approach. In addition, a modified Dega osteotomy [28, 29] was performed through a Smith-Petersen approach. Postoperatively, the patient was placed in a left lower extremity hip spica cast and remained nonweight bearing for six weeks. The cast was removed six weeks after surgery and physiotherapy was initiated. At ten months follow-up, she had persistent weakness of her abductors and hardware related pain over her left proximal femur. Pelvic radiographs showed good femoral head coverage (CEA 35 degrees) and a healed osteotomy (Figure 2(e)). However, partial osteonecrosis of the femoral head was noted. At one-year follow-up, her Trendelenburg gait persisted and she reported pain at the prominent hardware site. Her ROM and radiographs were unchanged from previous examination. At this time point, hardware removal was planned. At six months after removal, she was ambulating with a mild Trendelenburg. Her trochanteric pain was reported to be much better than before.

**Case 3**

**Figure 3:**
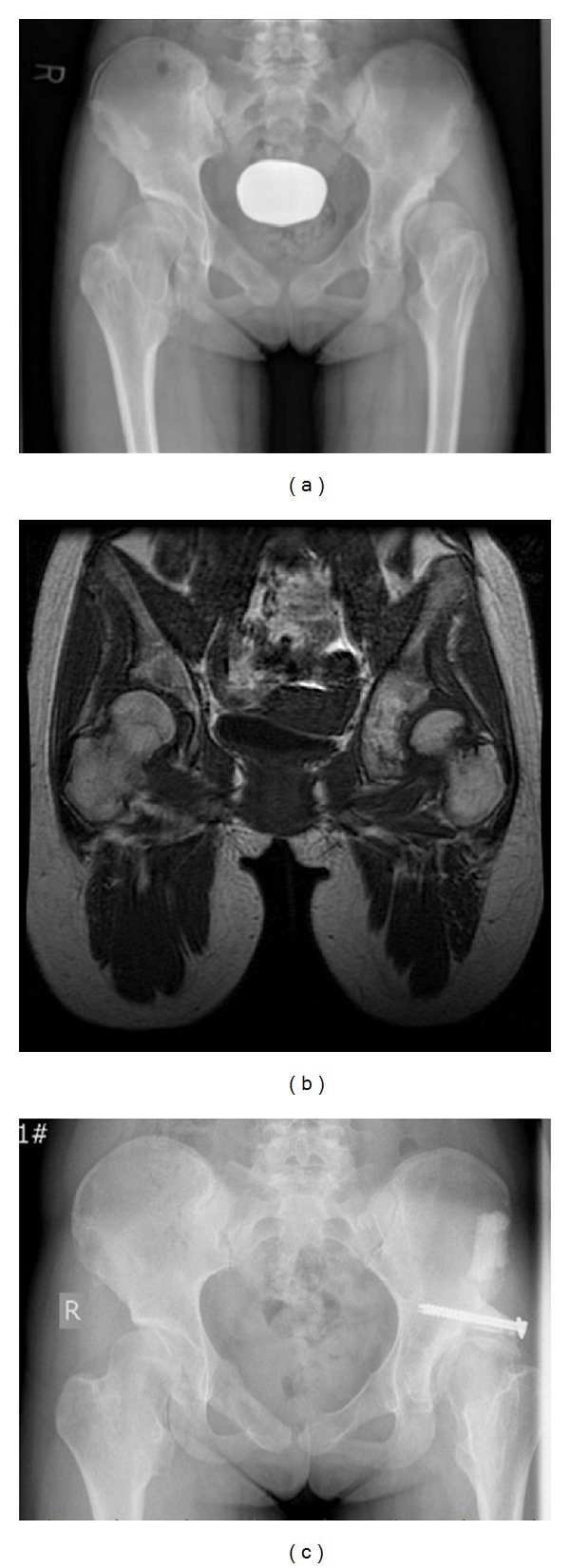
(a) Anteroposterior pelvic radiograph at initial presentation. (b) Magnetic resonance imaging showing a large sessile osteochondroma within the left acetabular fossa. (c) Anteroposterior radiograph of the pelvis 3 years after the left acetabular Shelf procedure and femoral neck osteoplasty.

This thirteen-year-old female known for HME was referred to our center for left groin pain with a locking sensation. She had undergone multiple previous surgeries in the lower extremities for excision of osteochondromas. On examination, she had limited flexion, abduction, and internal/external rotation. The radiographs showed bilateral acetabular dysplasia (CEA: left = −5 degrees/right = +10 degrees) with an increased left femoral neck width secondary to osteochondromas (Figure 3(a)). Magnetic resonance imaging showed a large sessile osteochondroma in the acetabular fossa (Figure 3(b)). The treating surgeon (M.L) has performed a left acetabular Shelf procedure and femoral neck osteoplasty through the anterior approach. The acetabular osteochondroma was not excised. Postoperatively, the patient was kept partial weight bearing for 6 weeks with ROM exercises as tolerated. At three-year follow-up, the patient reported no left hip pain and the ROM had improved significantly. Pelvic radiographs showed good femoral head coverage (CEA = 40 degrees) (Figure 3(c)).

## 4. Discussion

The presented cases have illustrated successful excision of femoral neck osteochondromas and treatment of acetabular dysplasia and poor femoral head coverage through three different surgical treatments. A strong relationship between HME and the occurrence of acetabular dysplasia has been reported in the literature [5]. It has been hypothesized that acetabular dysplasia occurs in HME secondary to biomechanical alterations in the hip joint. The osteochondromas can result in abnormal mechanical forces that may drive the dysplasia. It has also been hypothesized that coxa valga may contribute to the dysplasia [5, 26, 30]. There is no consensus in the current literature with respect to surgical treatment for hip osteochondromas when associated with acetabular dysplasia (Table 2). Malagón resected two femoral neck osteochondromas in two paediatric patients (8 and 9 years old) with acetabular dysplasia [5]. He also performed bilateral staged Chiari procedures along with varus femoral derotational osteotomies. Although satisfactory results were achieved, one patient had persisted hip pain and restricted ROM. Felix et al. resected bilateral femoral neck osteochondromas in a 12-year-old female patient who also had acetabular dysplasia. Bilateral staged resections, steel osteotomies, and proximal femoral varus osteotomies were performed through the posterior approach [10]. At 3 years of follow-up, no complications were reported. Shinozaki et al. resected a femoral neck osteochondroma in a 30-year-old male patient who had a dysplastic hip [16]. The authors resected the lesion through the anterior approach and posterior approach. A rotational osteotomy was also performed. At 6 weeks of follow-up, recurrence of hip subluxation was observed and the greater trochanter was transferred distally. Ofiram and Porat have reported a female patient (16 years old) who had an osteochondroma at the femoral neck (circumferential) and floor of acetabulum in association with acetabular dysplasia [9]. They excised the lesion through the anterior approach with intraoperative hip subluxation. No pelvic procedure was performed, and the patient remains asymptomatic at 3 years of follow-up. In conclusion, these case reports indicate that a combined approach of osteochondroma excision and pelvic osteotomy is feasible and tolerated well in the short term. One question that remains is whether or not early surgical excision of these lesions may prevent acetabular dysplasia. Jellicoe et al. [7] reported two paediatric patients (aged 9 and 11 years) with acetabular osteochondromas and acetabular dysplasia that were successfully treated with intraoperative excision of the lesions by surgically dislocating the hip. At 2 years of follow-up, although the patients had no symptoms, residual acetabular dysplasia and growth disturbance were found. The authors concluded that excision of osteochondromas appears not to prevent or improve acetabular dysplasia. Despite their conclusion, we still feel that early excision of the osteochondromas can prevent acetabular dysplasia when performed at young age. Theoretically speaking, if performed while the acetabulum still has significant remodelling potential, osteochondroma excision should affect acetabular development. Furthermore, acetabular dysplasia is often asymptomatic. Therefore, we strongly recommend a routine radiographic pelvic survey at the time of diagnosis of HME so that early detection of the osteochondroma can be made and treatment can be recommended. Unfortunately, there is no data available to recommend on the frequency of radiographic surveillance.

Osteochondromas can occur as solitary lesions in the proximal femur and these typically are not associated with acetabular dysplasia or coxa valga. However, many problems can arise from these lesions such as labral tears, nerve compression, hip dislocation, external snapping hip, and malignant transformation in 0.4–2% of patients [8, 13, 19, 20, 27]. A variety of surgical techniques have been reported in the literature for these solitary lesions without dysplasia (Table 3). The main concerns for surgical resection of femoral neck and peritrochanteric osteochondromas are exposure and femoral head vascularity. In our report (Case 2), we believe that the multiple surgeries around the hip might have put the femoral head blood supply at risk and contributed to the partial osteonecrosis. Siebenrock and Ganz have described the lateral approach to the hip with surgical hip dislocation to allow access and adequate exposure of the femoral neck while preserving the vascular supply [14]. They presented four adult patients with successful resection of femoral neck osteochondromas located in posterior, inferior, and anterior regions of the femoral neck. Li et al. [6] have echoed these results utilizing the same technique for the resection of a posteromedial femoral neck osteochondroma in one paediatric case. Using both anterior and posterolateral approaches, Ramos-Pascua et al. have successfully excised femoral neck osteochondromas in 6 patients without dislocating or subluxating the hip [19]. These patients had good to excellent results based on the Musculoskeletal Tumour Society (MSTS) scale. Tschokanow [15] reported on two adult cases of lesser trochanter osteochondromas in which one patient had an excision through the anterior approach and was complicated by femoral vein laceration and sciatic nerve palsy. The second patient underwent a two-staged procedure (through anterior and lateral approach) with no reported complications. Recently, Feely and Kelly have proposed the use of hip arthroscopy for excising small osteochondromas in the femoral neck [27]. Taken together, the literature review failed to conclude a uniform treatment for these lesions. Until further data is published, surgeons treating these lesions must carefully plan surgery such that a safe and adequate resection can be carried out in an effective manner utilizing the surgical approach they feel most comfortable with while paying particular attention to femoral head vascularity. In addition, the exact location of the lesion should be defined preoperatively to help develop a surgical plan and the use of intraoperative fluoroscopy can be helpful in localizing the lesion and in verifying adequate resection.

Few reports in the literature have described the occurrence of osteochondromas in the acetabulum. The majority of the reported cases underwent surgical hip dislocation/subluxation to excise the acetabular lesion (Table 4). The advantage of using the surgical dislocation approach is to gain full access to such lesions. Woodward et al. reported on two paediatric patients with acetabular and femoral neck osteochondromas excised through an anterior approach without the need for intraoperative hip dislocation [11]. Using hip arthroscopy, Bonnomet et al. successfully excised a small acetabular osteochondroma in an 11-year-old patient with HME [17]. In our report (Case 3), we did not excise the acetabular osteochondroma as it was large and sessile. Surgical excision of such large sessile lesions will result in significant acetabular cartilage and bone deficiency. Therefore, we chose to leave the acetabular lesion and treat the dysplasia by performing a Shelf augmentation procedure and excision of the femoral neck osteochondroma. Preoperative hinge abduction and the questionable quality of the remaining cartilage made periacetabular rotational osteotomy a suboptimal option.

In conclusion, the literature review failed to conclude a uniform treatment for patients with hip joint osteochondromas with or without hip dysplasia. The three reported cases illustrate the successful excision of femoral neck osteochondromas and treatment of acetabular dysplasia through three different surgical treatments. In HME patients, we believe that early excision of osteochondromas can prevent the occurrence of acetabular dysplasia. Therefore, we recommend a routine radiographic pelvic survey in HME patients at the time of diagnosis for early detection of osteochondromas in the hip. Our results suggest the need for a multi-institutional prospective study for the natural history of hip pain and arthrosis and the surgical treatment of hip joint osteochondromas and also for determining the frequency of radiographic pelvic surveys in HME patients.

## Figures and Tables

**Table 1:** Basic demographics and clinical data of three patients included in this report.

Patient	Age years	Gender	Presentation	Loss of range of motion	Radiographic finding	Location of osteochondroma	Procedure(s)
Case 1	15	Male	Left hip pain with prolonged activities.	Left hip: Flexion: 30° Abduction: 20°	Left hip subluxation secondary to femoral neck osteochondroma and acetabular dysplasia.	Anterior femoral neck.	Excision of osteochondroma and the Bernese periacetabular osteotomy through a modified Smith-Peterson approach.

Case 2	4	Female	Difficulty with movement of left hip noted by patient's mother.	Left hip: Internal rotation: 10° External rotation: 15° Flexion: 20° Abduction: 30°	(1) Left hip subluxation secondary to femoral neck osteochondroma and acetabular dysplasia. (2) Bilateral coxa valga.	Medial femoral neck and posterior intertrochanteric region.	At 4 years of age: proximal femur VDRO^1^ with excision of osteochondroma and application of DHS^2^ through lateral approach. At 8 years of age: left modified Dega osteotomy through anterior approach, removal of DHS, VDRO^1^, and application of LCP^3^ through lateral approach. At 9 years of age: removal of LCP.

Case 3	13	Female	Left hip pain with activity and sensation of locking.	Left hip: Internal rotation: 15° External rotation: 10° Abduction: 20°	Left hip subluxation secondary to femoral neck, acetabular osteochondroma, and acetabular dysplasia.	Acetabular fossa and anterior femoral neck.	Excision of the femoral neck osteochondroma and Shelf procedure through anterior approach.

^1^Varus derotational osteotomy. ^2^Dynamic hip screw. ^3^Locking compression plate.

**Table 2:** Literature review. Surgical treatments for hip osteochondroma with acetabular dysplasia in previous studies.

Author and date	Number of patients	Age	Gender	Location of the lesion	Procedure	Follow-up period	Complications
Malagón 2001 [5]	Two	Nine years	Male	Medial femoral neck.	(1) Right femoral varus osteotomy. (2) Bilateral staged Chiari procedures.	Four years	Persistent hip pain and limited ROM^1^.
		Eight years	Male	Femur neck (the exact location is not specified).	Bilateral proximal varus femur osteotomy.	Not specified	Not specified.

Felix et al., 2000 [10]	One	12 years	Female	Bilateral medial femoral neck.	(1) Bilateral staged excision through the posterior approach and VDRO^2^. (2) Bilateral staged steel osteotomy.	Two years	Not reported.

Shinozaki et al., 1998 [16]	One	30 years	Male	Femoral neck (the exact location is not specified).	Excision through the anterior iliofemoral and posterior approach. Rotational acetabular osteotomy was performed.	Two years	Recurrence of subluxation at 6 weeks after surgery. Greater trochanter distal transfer was then performed.

Jellicoe et al., 2009 [7]	Two	Nine years	Female	Circumferential femoral neck and floor of acetabulum.	Excision through anterolateral approach and surgical hip dislocation. No pelvic osteotomy was performed.	Two years	Not reported.
		11 years	Male	Cotyloid foramen.	Excision through transtrochanteric approach and surgical hip dislocation. No pelvic osteotomy was performed.	Three years	

Ofiram and Porat, 2004 [9]	One	16 years	Female	Circumferential at the femoral neck and also at the acetabular floor.	Excision through Smith-Peterson approach and intraoperative hip subluxation. No pelvic osteotomy was performed.	Three years	Not reported.

**Table 3:** Literature review. Surgical treatments for solitary proximal femoral osteochondroma in previous studies.

Author and date	Number of patients	Age in years	Gender	Location of the lesion	Procedure	Follow-up period	Complications
Yu et al., 2010 [13]	One	39	Male	Posterior FN^1^	Excision through a posterior approach	22 months	Not reported.

Siebenrock and Ganz, 2002 [14]	Four	26 30 20 39	Male Female Male Female	(1) Posterior inferior FN (2) Anterior, inferior, and posterior FN (3) Anteroinferior FN (4) Inferior FN	Excision through lateral approach and digastric trochanteric osteotomy followed by (i) surgical hip dislocation in two patients, (ii) hip subluxation in the other two patients	18–48 month	One patient had intermittent pain in greater trochanter area on follow-ups.

Tschokanow, 1969 [15]	Two	33 36	Male Male	Lesser trochanter Lesser trochanter	Anterior approach Anterior and lateral approach (staged procedures with 2-month interval)	Not specified	Femoral vein injury and sciatic nerve palsy. Postoperative wound infection. Not reported.

Feeley and Kelly, 2009 [27]	One	37	Female	Anterior FN	Excision by hip arthroscopy	Six months	Not reported.

Hussain et al., 2010 [25]	One	24	Male	Posterior FN	Excision through posterolateral approach	Seven months	Persisted pain due to FA^2^ impingement.

Ramos-Pascua et al., 2012 [19]	Six	20 45 50 66 28 29	Male Male Male Female Female Male	Medial FN Anterior FN Medial FN Medial FN Anterior FN Anterior FN	Excision through anterior approach in 3 patients, and by posterolateral approach on the other 3 patients.	From 2 to 20 years	One patient had basicervical fracture and was treated successfully with no sequelae.

Li et al., 2012 [6]	One	11	Male	Medial and posterior FN	Excision through a surgical hip dislocation (digastric approach)	Seven years	Not reported.

Jones and Kinninmonth, 2005 [8]	One	18		Posteroinferior FN	Excision through posterior approach	Not specified	?

Liu et al., 2010 [23]	One	Six	Male	Posterior FN	Excision through lateral approach	Four years	Not reported.

Learmonth and Raymakers, 1993 [12]	One	13	Female	At the femoral epiphyseal plate	Excision through Smith-Peterson approach	Not specified	?

Magid et al., 1996 [24]	One	14	Female	FN (exact location is not specified)	Excision through posterior approach	Nine months	Non reported

Muzaffar et al., 2012 [18]	One	22	Female	Base of FN	Excision through posterolateral approach	Not specified	?

^1^Femoral neck. ^2^Femoroacetabular.

**Table 4:** Literature review. Surgical treatments for acetabular osteochondroma in previous studies.

Author and date	Number of patients	Age	Gender	Location of the lesion	Procedure	Follow-up period	Complications
Ofiram and Porat, 2004 [9]	One	16 years	Female^1^	Circumferential at the femoral neck also at the acetabular floor	Excision through Smith-Peterson approach and intraoperative hip subluxation.	Three years	Not reported

Woodward et al., 1999 [11]	Two	Three years	Male	Base of acetabulum and femoral neck	Excision through anterior approach followed by hip spica for 6 weeks.	Three months	Not reported
		11 years	Female	Inferomedial acetabulum and anterior femoral neck	Excision through anterior approach.	14 months	

Bonnomet et al., 2001 [17]	Two	11 years Nine years	Male Female	Acetabular fossa Acetabular fossa	Excision by hip arthroscopy technique. Excision by hip arthroscopy technique.	Three years Two years	Not reported

Ettl et al., 2006 [22]	Two	Eight years	Male	Acetabular floor	Excision though anterolateral approach and hip subluxation. The patient also had VDRO^2^ to correct the coxa valga.	Two years	Not reported

Jellicoe et al., 2009 [7]	Two	Nine years	Female^1^	Circumferential femoral neck and floor of acetabulum	Excision through anterolateral approach and surgical hip dislocation.	Two years	Not reported
		11 years	Male^1^	Cotyloid foramen	Excision through transtrochanteric approach and surgical hip dislocation.	Three years	

Bracq et al., 1987 [21]	One	Three years	Female	Base of the acetabulum	Excision through the Hueter anterior approach and surgical hip dislocation.	Three years	Not reported

^1^These patients have had associated acetabular dysplasia in the affected hip. ^2^Varus derotational osteotomy.

## References

[1] Peterson HA (1989). Multiple hereditary osteochondromata. *Clinical Orthopaedics and Related Research*.

[2] Shapiro F, Simon S, Glimcher MJ (1979). Hereditary multiple exostoses. Anthropometric, roentgenographic, and clinical aspects. *The Journal of Bone and Joint Surgery. American*.

[3] Schmale GA, Conrad EU, Raskind WH (1994). The natural history of hereditary multiple exostoses. *The Journal of Bone and Joint Surgery. American*.

[4] Cheung PK, McCormick C, Crawford BE, Esko JD, Tufaro F, Duncan G (2001). Etiological point mutations in the hereditary multiple exostoses gene EXT1: a functional analysis of heparan sulfate polymerase activity. *American Journal of Human Genetics*.

[5] Malagón V (2001). Development of hip dysplasia in hereditary multiple exostosis. *Journal of Pediatric Orthopaedics*.

[6] Li M, Luettringhaus T, Walker KR, Cole PA (2012). Operative treatment of femoral neck osteochondroma through a digastric approach in a pediatric patient: a case report and review of the literature. *Journal of Pediatric Orthopaedics B*.

[7] Jellicoe P, Son-Hing J, Hopyan S, Thompson GH (2009). Surgical hip dislocation for removal of intraarticular exostoses: report of two cases. *Journal of Pediatric Orthopaedics*.

[8] Jones BG, Kinninmonth AWG (2005). Low-energy hip dislocation in the young. *Journal of Trauma*.

[9] Ofiram E, Porat S (2004). Progressive subluxation of the hip joint in a child with hereditary multiple exostosis. *Journal of Pediatric Orthopaedics B*.

[10] Felix NA, Mazur JM, Loveless EA (2000). Acetabular dysplasia associated with hereditary multiple exostoses: a case report. *The Journal of Bone and Joint Surgery. British*.

[11] Woodward MN, Daly KE, Dodds RDA, Fixsen JA (1999). Subluxation of the hip joint in multiple hereditary osteochondromatosis: report of two cases. *Journal of Pediatric Orthopaedics*.

[12] Learmonth DJA, Raymakers R (1993). Osteochondroma of the femoral neck secondary to a slipped upper femoral epiphysis. *Archives of Orthopaedic and Trauma Surgery*.

[13] Yu K, Meehan JP, Fritz A, Jamali AA (2010). Osteochondroma of the femoral neck: a rare cause of sciatic nerve compression. *Orthopedics*.

[14] Siebenrock K-A, Ganz R (2002). Osteochondroma of the femoral neck. *Clinical Orthopaedics and Related Research*.

[15] Tschokanow K (1969). 2 cases of osteochondroma of the femur neck. *Beitrage zur Orthopadie und Traumatologie*.

[16] Shinozaki T, Watanabe H, Inoue J, Ogiwara T (1998). Rotational acetabular osteotomy in a dysplastic hip with femoral neck osteochondromas. *Orthopedics*.

[17] Bonnomet F, Clavert P, Abidine FZ, Gicquel P, Clavert JM, Kempf JF (2001). Hip arthroscopy in hereditary multiple exostoses: a new perspective of treatment. *Arthroscopy*.

[18] Muzaffar N, Bashir N, Baba A, Ahmad A, Ahmad N (2012). Isolated osteochondroma of the femoral neck presenting as hip and leg pain. A case study. *Ortopedia, Traumatologia, Rehabilitacja*.

[19] Ramos-Pascua L, Sánchez-Herráez S, Alonso-Barrio J, Alonso-León A (2012). Solitary proximal end of femur osteochondroma. An indication and result of the en bloc resection without hip luxation. *Revista Española de Cirugía Ortopédica y Traumatología*.

[20] Inoue S, Noguchi Y, Mae T, Rikimaru S, Hotokezaka S (2005). An external snapping hip caused by osteochondroma of the proximal femur. *Modern Rheumatology*.

[21] Bracq H, Guibert L, Fremond B (1987). A case of exostosis of the base of the acetabulum in a child with multiple exostoses. *Revue de Chirurgie Orthopedique et Reparatrice de l’Appareil Moteur*.

[22] Ettl V, Siebenlist S, Rolf O, Kirschner S, Raab P (2006). Intraacetabular localisation of an osteochondroma causing subluxation of the hip joint—a rare entity in children with multiple hereditary exostoses. *Zeitschrift für Orthopädie und Ihre Grenzgebiete*.

[23] Liu ZJ, Zhao Q, Zhang LJ (2010). Extraskeletal osteochondroma near the hip: a pediatric case. *Journal of Pediatric Orthopaedics B*.

[24] Magid D, Sponseller PD, McCarthy E (1996). Intoeing in a 14-year-old girl. *Clinical Orthopaedics and Related Research*.

[25] Hussain W, Avedian R, Terry M, Peabody T (2010). Solitary osteochondroma of the proximal femur and femoral acetabular impingement. *Orthopedics*.

[26] Porter DE, Benson MK, Hosney GA (2001). The hip in hereditary multiple exostoses. *The Journal of Bone and Joint Surgery. British*.

[27] Feeley B, Kelly B (2009). Arthroscopic management of an intraarticular osteochondroma of the hip. *Orthopedic Reviews*.

[28] Al-Ghamdi A, Rendon JS, Al-Faya F, Saran N, Benaroch T, Hamdy RC (2012). Dega osteotomy for the correction of acetabular dysplasia of the hip: a radiographic review of 21 cases. *Journal of Pediatric Orthopaedics*.

[29] Mubarak SJ, Valencia FG, Wenger DR (1992). One-stage correction of the spastic dislocated hip. Use of pericapsular acetabuloplasty to improve coverage. *The Journal of Bone and Joint Surgery. American*.

[30] El-Fiky TAM, Chow W, Li YH, To M (2009). Hereditary multiple exostoses of the hip. *Journal of Orthopaedic Surgery*.

